# B_0_ Correction for 3T Amide Proton Transfer (APT) MRI Using a Simplified Two-Pool Lorentzian Model of Symmetric Water and Asymmetric Solutes

**DOI:** 10.3390/tomography8040165

**Published:** 2022-08-01

**Authors:** Yibing Chen, Xujian Dang, Benqi Zhao, Zhuozhao Zheng, Xiaowei He, Xiaolei Song

**Affiliations:** 1 Xi’an Key Laboratory of Radiomics and Intelligent Perception, School of Information Sciences and Technology, Northwest University, Xi’an 710069, China; cyb@stumail.nwu.edu.cn (Y.C.); dangxujian@stumail.nwu.edu.cn (X.D.); hexw@nwu.edu.cn (X.H.); 2 Department of Radiology, Beijing Tsinghua Changgung Hospital, Beijing 102218, China; zbqa01279@btch.edu.cn (B.Z.); zzza00509@btch.edu.cn (Z.Z.); 3 Center for Biomedical Imaging Research, Department of Biomedical Engineering, Tsinghua University, Beijing 100084, China

**Keywords:** amide proton transfer (APT) MRI, B_0_ inhomogeneity correction, brain tumors, Lorentzian fitting, chemical exchange saturation transfer (CEST) MRI

## Abstract

Amide proton transfer (APT)-weighted MRI is a promising molecular imaging technique that has been employed in clinic for detection and grading of brain tumors. MTR_asym_, the quantification method of APT, is easily influenced by B_0_ inhomogeneity and causes artifacts. Current model-free interpolation methods have enabled moderate B_0_ correction for middle offsets, but have performed poorly at limbic offsets. To address this shortcoming, we proposed a practical B_0_ correction approach that is suitable under time-limited sparse acquisition scenarios and for B_1_ ≥ 1 μT under 3T. In this study, this approach employed a simplified Lorentzian model containing only two pools of symmetric water and asymmetric solutes, to describe the Z-spectral shape with wide and ‘invisible’ CEST peaks. The B_0_ correction was then performed on the basis of the fitted two-pool Lorentzian lines, instead of using conventional model-free interpolation. The approach was firstly evaluated on densely sampled Z-spectra data by using the spline interpolation of all acquired 16 offsets as the gold standard. When only six offsets were available for B_0_ correction, our method outperformed conventional methods. In particular, the errors at limbic offsets were significantly reduced (*n* = 8, *p* < 0.01). Secondly, our method was assessed on the six-offset APT data of nine brain tumor patients. Our MTR_asym_ (3.5 ppm), using the two-pool model, displayed a similar contrast to the vendor-provided B_0_-orrected MTR_asym_ (3.5 ppm). While the vendor failed in correcting B_0_ at 4.3 and 2.7 ppm for a large portion of voxels, our method enabled well differentiation of B_0_ artifacts from tumors. In conclusion, the proposed approach could alleviate analysis errors caused by B_0_ inhomogeneity, which is useful for facilitating the comprehensive metabolic analysis of brain tumors.

## 1. Introduction

As a type of chemical exchange saturation transfer (CEST) imaging [1,2,3], amide proton transfer (APT) imaging is a promising, non-invasive molecular MRI technique that can detect endogenous mobile proteins and peptides in tissue [4,5]. Numerous institutions worldwide have demonstrated that APT imaging adds important value to the standard clinical MRI sequences in brain tumor diagnoses, such as finding biomarkers, monitoring tumor progression and response to treatment, grading gliomas, etc. [6,7,8,9]. Due to the asymmetric nature of CEST signals, asymmetry analysis of the magnetization transfer ratio (i.e., MTR_asym_) is employed to quantify APT MRI, which equates to the subtraction of normalized saturation signals at two symmetric offsets around the water frequency (i.e., 0 ppm). MTR_asym_ is susceptible to B_0_ inhomogeneity, and B_0_ artifacts interfere with the identification and analysis of brain tumors. Therefore, B_0_ inhomogeneity correction is important for the quantification and clinical applications of APT imaging.

Limited by the scan time, in the clinical APT protocol, a few saturation offsets are acquired around +3.5 ppm and −3.5 ppm for the post-processing of B_0_ inhomogeneity correction, instead of real-time B_0_ correction being performed during acquisition [10,11,12,13]. The most commonly used post-processing correction methods are interpolation-based methods, which include two steps. First, densely sampled signals with intervals of 0.1 ppm are interpolated from sparsely acquired signals using spline or other interpolated methods [14,15,16,17,18]. Second, the B_0_ inhomogeneity is corrected through a B_0_ inhomogeneity (ΔB_0_) map of the same image geometry. A ΔB_0_ map can be obtained using water saturation shift referencing (WASSR) [19,20], Dixon [21], or Lorentzian-based methods [22].

Interpolation-based methods require high-frequency resolutions and signals close to the water frequency to provide line shape and adequate neighborhood information. For example, Debnath et al. found that linear interpolation was suitable for the B_0_ correction of APT data (B_1_ = 2 μT) acquired at 64 offsets (−14~14 ppm with 0.5 ppm intervals) [15]. However, as it is limited by scan time, when using the clinical APT protocol, only a few saturation offsets can be sampled around ±3.5 ppm. Due to insufficient data acquisition, interpolation-based methods perform poorly at limbic offsets, which means the APT protocol typically only provides MTR_asym_ (3.5 ppm). Therefore, the acquisition signals of the APT protocol are not actually fully used, meaning some important metabolites are discarded, such as the fast exchange amine (2.7 ppm) and semi-solid macromolecules (4.3 ppm) [21].

The a priori introduction of a line-shape constraint may compensate for the disadvantages caused by insufficient data acquisition in the B_0_ correction process. Zhou et al. reported Z-spectral line-shapes of brain tumor patients (B_0_ = 3T, B_1_ =2 μT) in their APT imaging review, which indicates that Z-spectral line-shapes are determined by four effects, i.e., direct water saturation (DS), semi-solid magnetization transfer (MT), CEST and the relayed nuclear Overhauser effect (NOE) [9]. The four effects can be simply divided into two components according to whether the effect is symmetry around water frequency, i.e., a symmetric component including DS, and an asymmetric component including MT, CEST and NOE. Lee et al. proposed a model-based CEST analysis method, which also separated Z-spectra into symmetric and asymmetric parts and used a Lorentzian model to fit the symmetric part [23]. Therefore, limited by few acquisition offsets, a two-pool Lorentzian model may be the correct choice to describe Z-spectral line shapes under 3T with high B_1_, in which one pool fits the symmetric water and another pool fits all asymmetric solutes.

Herein, we propose a practical B_0_ correction approach for use in the most popular six-offset acquisition protocol [24]. Using this approach, we employed a two-pool (symmetric water and asymmetric solutes) Lorentzian model to fit the Z-spectral line shape of human brains at 3T with B_1_ = 2 μT, in a voxel-by-voxel manner. We evaluated our method using in vivo APT data acquired from the brains of healthy volunteers and tumor patients. The contributions of the present study are as follows: (a) In theory, we propose a simplified two-pool Lorentzian model that is suitable to describe the Z-spectral line shape of human brains under 3T with B_1_ ≥ 1 μT. The reduced number of model parameters allowed for fitting using less frequency offsets, i.e., six offsets as in the popular APT protocol. (b) Compared with conventional model-free interpolation, the proposed method could better recover the Z-spectral signals and improve B_0_ correction performance, especially for limbic offsets.

## 2. Theory

**Figure 1:**
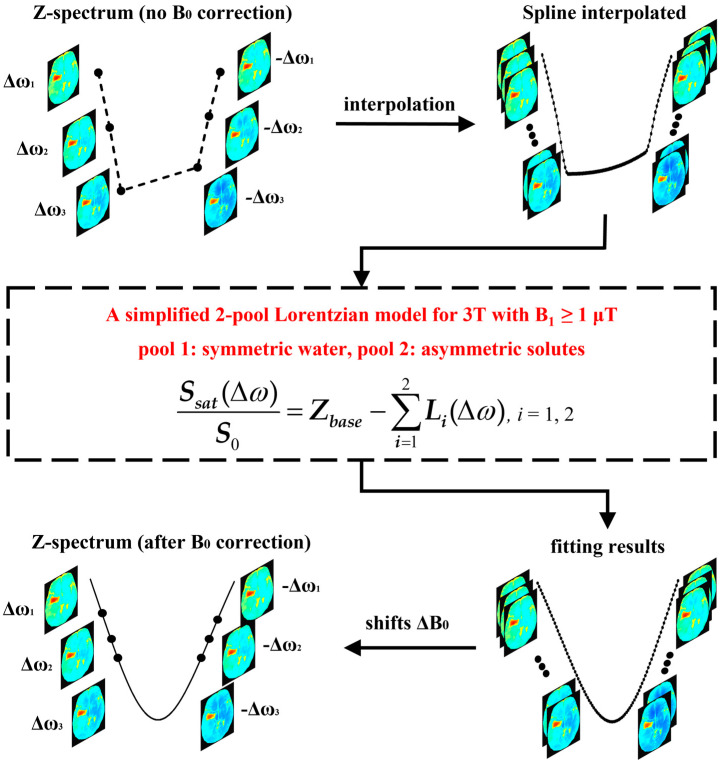
The flowchart of the proposed 2-pool-Lorentzian-based B_0_ correction method.

To robustly describe the Z-spectral line shape under 3T with high B_1_ (2 μT), a simplified two-pool Lorentzian model was chosen as an a priori line shape to fit the spline-interpolated initial Z-spectra (ranging ±2.5~±4.5 ppm). Given the small number of acquired offsets and the broadening of the peaks, we used the simplest standard to construct our two-pool model, i.e., symmetric water was considered as one pool, and all asymmetric solutes were considered as another pool, which included a large portion of semi-solid macromolecules, a relayed nuclear Overhauser effect (NOE), amides and other metabolites. Equations (1) and (2) present the model function of our two-pool Lorentzian method.

(1) $$\frac{S_{sat}\left(\Delta \omega \right)}{S_{0}}=Z_{base}-\sum_{i=1}^{2}L_{i}\left(\Delta \omega \right)$$

(2) $$L_{i}\left(\Delta \omega \right)=\frac{A_{i}}{1+{\left(\Delta \omega -\Delta_{i}\right)}^{2}/{\left(0.5W_{i}\right)}^{2}}$$

where *i* = 1 (symmetric water), 2 (asymmetric solutes); the parameter *Z_base_* is used to correct for a constant signal reduction; *L_i_* represents a Lorentzian line with a central offset (Δ*_i_*), peak full width at half maximum (FWHM, *W_i_*), and peak amplitude (*A_i_*); *S_sat_*(Δ*ω*)/*S*_0_ is the normalized Z-spectrum. Based on previous studies [25,26] and our experiences, the starting points and boundaries of the fitting parameters are shown in Table 1. The flowchart of the proposed B_0_ correction procedure is illustrated in Figure 1.

## 3. Materials and Methods

### 3.1. B_0_ inhomogeneity Correction

The APT data were normalized by S_0_ (−1560 ppm) without saturation pulse, and smoothed using a 4 × 4 median filter according to the acquisition matrix. The proposed 2-pool Lorentzian-based B_0_ correction method was used in a voxel-by-voxel manner (Figure 1). For each voxel, a 6-offset Z-spectrum was interpolated, generating a fine Z-spectrum with interval of 0.1 ppm. In this study, the cubic-spline method, implemented by MATLAB 2021, a function spline, was employed for interpolation, because it shows less B_0_ artifacts on MTR_asym_ (2.7 ppm) than linear and cubic-Hermite interpolation (Figure S1 in the Supplementary Materials). Then, the 2-pool (i.e., symmetric water and asymmetric solutes) Lorentzian model was utilized to fit the line shape of the interpolated Z-spectrum. Finally, after fitting, the Z-spectrum was shifted along the frequency dimension to the correct position, according to a B_0_ inhomogeneity (ΔB_0_).

### 3.2. MTR_asym_ Quantification of APT

After voxel-by-voxel B_0_ correction, magnetization transfer ratio asymmetry analysis (i.e., MTR_asym_) was employed to quantify APT, which is defined as Equation (3).

(3) $${\mathrm{MTR}}_{\mathrm{asym}}\left(\Delta \omega_{j}\right)=\frac{S_{sat}(-\Delta \omega_{j})-S_{sat}(\Delta \omega_{j})}{S_{0}}$$

where Δ*ω_j_* represents an offset.

### 3.3. Comparison Methods

The vendor, the cubic-spline interpolation-based method (spline) and the 1-pool Lorentzian-based method (1-pool) were employed as comparison methods. The vendor represents the correction results provided by Philips Healthcare [27]. The 1-pool Lorentzian-based method is similar to the proposed 2-pool Lorentzian-based method, merely replacing the 2-pool (water and solutes) Lorentzian model with the 1-pool (water) Lorentzian model.

### 3.4. Datasets

In this study, first, we recruited 4 healthy volunteers (2 males and 2 females, aged 22 ± 3.4 years) and 4 brain tumor patients (3 males and 1 female, aged 53.5 ± 14.8 years). From these 8 subjects, densely sampled 16-offset APT data were acquired (−6.7, -5.9, ±5.1, ±4.3, ±3.5, ±2.7, ±1.9, ±1.1 and ±0.3 ppm) to validate the accuracy of our method. Then, we recruited 9 brain tumor patients (3 males and 6 females, aged 54.4 ± 18.6 years), from whom we acquired 6-offset APT data to enable further comparisons with the vendor. Note that the 16-offset APT protocol was modified by us; therefore, it did not include B_0_ correction results from the vendor. For our method and comparison methods, 6-offset images (±2.7, ±3.5 and ±4.3 ppm) were extracted from 16-offset APT data. The B_0_ correction results using the spline interpolation-based method of 16-offset images were considered to be the gold standard. Of the 13 brain tumor patients, 8 had glioblastomas, 4 had meningiomas and 1 had a metastatic brain tumor from lung cancer.

The study protocol was approved by the institutional review board, and written informed consent was obtained from each subject. MR experiments were performed on a 3T Ingenia MRI system (Philips Healthcare) with a 32-channel phase array coil, using an APT sequence and a turbo spin echo readout. For brain tumor patients, APT data were acquired on the slice centered at the largest areas of the tumors shown on T2w images. The imaging parameters for APT sequences were as follows: T_sat_ = 2 s, B_1_ = 2 μT, echo time = 8.3 ms, repetition time = 5 s, slice thickness = 7 mm and field-of-view = 220 × 201 mm^2^ with an acquisition voxel size = 2.5 × 2.5 × 7 mm^3^. ΔB_0_ maps were generated using the 3-echo Dixon. Multi-slice T2w images and Gd-T1w images were acquired with a 5 mm slice thickness.

### 3.5. Evaluation Metrics

Using the gold standard as described in Section 3.4, Z-spectra errors and MTR_asym_ errors were employed to evaluate the accuracy of B_0_ correction, which are defined as Equations (4) and (5).

Z-spectra errors = |Z-spectra(2-pool/comparisons) − Z-spectra(gold standard)|,(4)

MTR_asym_ errors = |MTR_asym_(2-pool/comparisons) − MTR_asym_(gold standard)|. (5)

One-tailed, paired Student’s *t*-tests were used to evaluate the differences between two groups in this study, which were considered to be statistically significant when *p* < 0.05.

## 4. Results

### 4.1. Accuracy Evaluation

**Figure 2:**
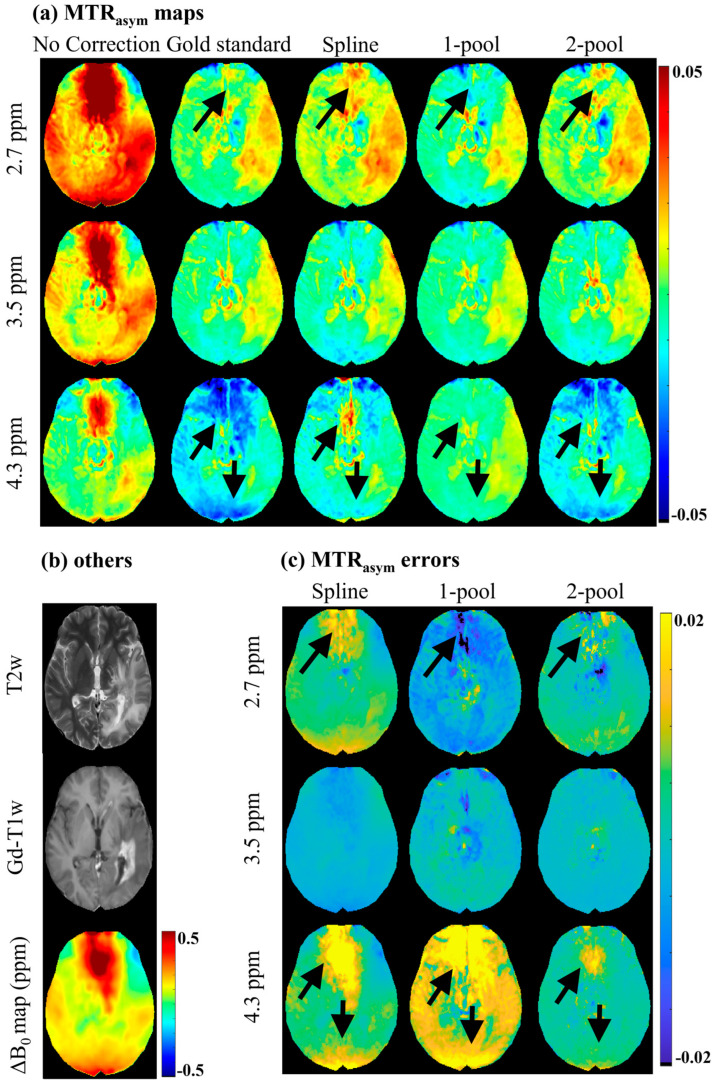
MTR_asym_ maps and MTR_asym_ error maps of a representative brain tumor patient. (**a**) MTR_asym_ maps without B_0_ correction, gold standard, and MTR_asym_ maps corrected using spline, 1-pool Lorentzian and 2-pool Lorentzian methods; (**b**) T2w, Gd-T1w and ΔB_0_ map; (**c**) the corresponding MTR_asym_ error maps with gold standard.

Figure 2 shows the MTR_asym_ maps and the corresponding error maps of a representative brain tumor patient. As seen in Figure 2a, before B_0_ correction, MTR_asym_ maps had severe B_0_ artifacts, which would have influenced the identification and analysis of tumors (as shown in Figure 2b), especially for 2.7 ppm. The spline-based correction method alleviated some B_0_ artifacts; however, in the regions with high B_0_ inhomogeneity (ΔB_0_ > 0.5 ppm), the artifacts still appeared on MTR_asym_ (2.7 ppm) and MTR_asym_ (4.3 ppm). The one-pool Lorentzian-based method seemed to eliminate artifacts in regions with high B_0_ inhomogeneity, but generated wrong MTR_asym_ (4.3 ppm) maps, which were quite different from the gold standard. Compared with the maps generated using the spline and one-pool methods, the three MTR_asym_ maps corrected using the proposed two-pool Lorentzian-based method not only were more similar to the gold standard, but also had fewer B_0_ artifacts. This can also be validated by the MTR_asym_ error maps, shown in Figure 2c. Using our method, there were fewer MTR_asym_ errors of limbic offsets than when using the spline and one-pool methods, especially in the regions with relatively high B_0_ inhomogeneity. The MTR_asym_ maps and MTR_asym_ error maps of a representative healthy volunteer are shown in Figure S2 in the Supplementary Materials.

**Figure 3:**
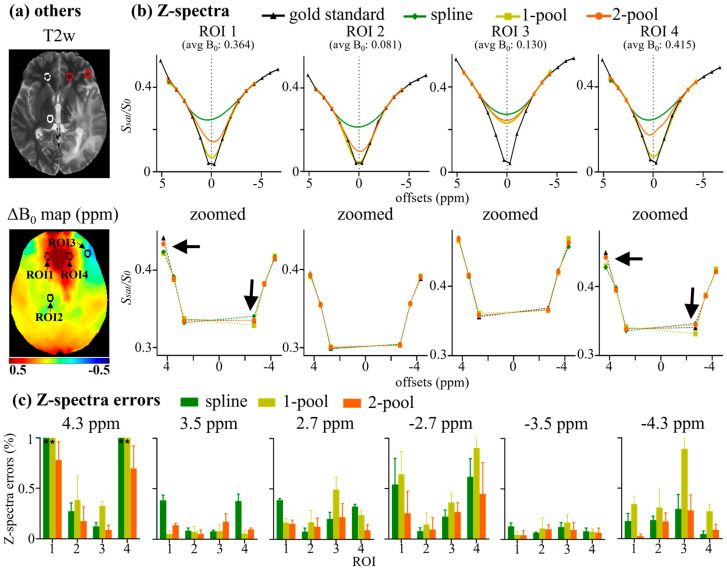
ROI analysis of a representative tumor patient. (**a**) Four circle ROIs (radius = 5 voxels) with different B_0_ inhomogeneity, shown on T2w and ΔB_0_ map; (**b**) mean Z-spectra of ROIs, including gold standard, and Z-spectra corrected using spline, 1-pool Lorentzian, and 2-pool Lorentzian methods; (**c**) the corresponding Z-spectra error with gold standard.

The corresponding region-of-interest (ROI) analyses of the representative tumor patient are shown in Figure 3. Four circle ROIs (radius = 5 voxels) with different B_0_ inhomogeneities (ΔB_0_) are displayed on T2w and a ΔB_0_ map (Figure 3a). As seen in Figure 3b, for the ROIs with relatively low ΔB_0_ (~0.1 ppm), i.e., ROI 2 and 3, the line shape of two ROIs were similar to each other, showing almost a direct line from 2.7 to 4.3 ppm and an almost ‘invisible’ peak at −3.5 ppm. Using the corrected Z-spectra with low ΔB_0_ as the internal standard, we found that the Z-spectral line-shape of high ΔB_0_ (~0.4 ppm) ROIs, corrected using our method, were similar to the internal standard. In contrast, Z-spectra corrected using the spline showed more obvious peaks at 3.5 ppm. Furthermore, Figure 3c shows that the Z-spectra errors using our method were nearly less than 0.5%, and were also less than those generated using the spline and one-pool methods. Similarly, four ROIs, the mean Z-spectra of ROIs and the corresponding Z-spectra errors of the representative healthy volunteer are shown in Figure S3 in the Supplementary Materials.

**Figure 4:**
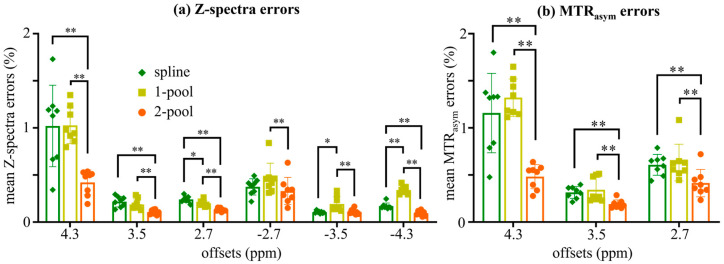
Statistical analysis of 8 subjects (4 healthy volunteers and 4 brain tumor patients). (**a**) The statistical results of mean Z-spectra errors corrected using spline, 1-pool Lorentzian and 2-pool Lorentzian methods at 6 offsets. (**b**) The statistical results of mean MTR_asym_ error corrected using spline, 1-pool Lorentzian and 2-pool Lorentzian methods; * *p* < 0.05; ** *p* < 0.01.

We statistically analyzed the Z-spectra errors and MTR_asym_ errors of eight subjects (four healthy volunteers and four brain tumor patients). The results are shown in Figure 4, which were consistent with the experimental results from the representative subject (Figure 2 and Figure 3). As seen in Figure 4a, the spline and one-pool methods performed poorly at the limbic offsets, especially for 4.3 ppm, for which the Z-spectra errors of those two methods were around 1%. In contrast, the Z-spectra error from our method for 4.3 ppm was half of that caused by the spline and one-pool methods (~0.5%). For all the offsets, the Z-spectra errors from our method were the lowest among the three correction methods. From Figure 4b, for all the offsets, the MTR_asym_ errors from our method were less than 0.5% and were significantly lower than those from the spline and one-pool methods (*p* < 0.01). In particular, our method dramatically decreased the MTR_asym_ error of 4.3 ppm.

As suggested by the experiments with the gold standard, and especially by the statistical results, our method reduced the Z-spectra error and MTR_asym_ error more effectively than the interpolation-based method (i.e., spline) and the one-pool Lorentzian-based method (i.e., one-pool).

### 4.2. Comparison with Vendor

**Figure 5:**
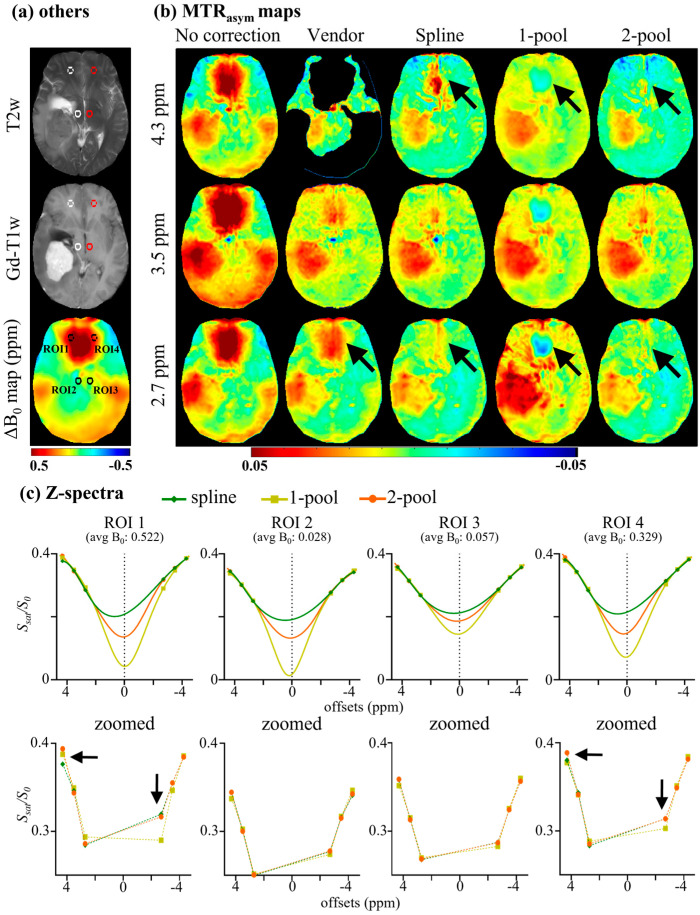
MTR_asym_ maps and ROI analysis of a meningioma patient. (**a**) Four circle ROIs (radius = 5 voxels) with different B_0_ inhomogeneities, shown on T2w, Gd-T1w, and ΔB_0_ map; (**b**) MTR_asym_ maps without B_0_ correction and MTR_asym_ maps corrected using vendor, spline, 1-pool Lorentzian and 2-pool Lorentzian methods; (**c**) mean Z-spectra of ROIs.

To compare our method with the vendor, the six-offset APT data of nine brain tumor patients were acquired, as described in Section 3.4. Figure 5 displays the MTR_asym_ maps and corresponding ROI analyses of a representative meningioma patient. Four circle ROIs (radius = 5 voxels) with different ΔB_0_ are shown in T2w, Gd-T1w and a ΔB_0_ map (Figure 5a). Figure 5b shows MTR_asym_ without B_0_ correction and MTR_asym_ corrected using the vendor, spline, one-pool Lorentzian and two-pool Lorentzian methods. As seen in Figure 5b, the correction results from the vendor still showed obvious B_0_ artifacts on MTR_asym_ maps, even for MTR_asym_ (3.5 ppm). Due to the interference of B_0_ artifacts, we could not identify the tumor region without Gd-T1w and T2w. The one-pool-Lorentzian-based method also had severe B_0_ artifacts, like the vendor. The spline method and our two-pool-Lorentzian-based method efficiently reduced B_0_ artifacts, which could help in the identification of tumors. However, the spline-based method still displayed some B_0_ artifacts at limbic offsets, especially at 4.3 ppm, while our two-pool-Lorentzian-based method also reduced those artifacts. Similar to the method used in Section 4.1, the corrected Z-spectra of low ΔB_0_ ROIs (<0.1 ppm) were considered to be the internal standard for comparison with the corrected Z-spectra of high ΔB_0_ ROIs (>0.3 ppm). As seen in Figure 5c, the corrected Z-spectral line shapes observed using our method were close to the internal standard, while the use of the spline method caused a peak at 3.5 ppm, and the line shapes observed using the one-pool method were too symmetric.

**Figure 6:**
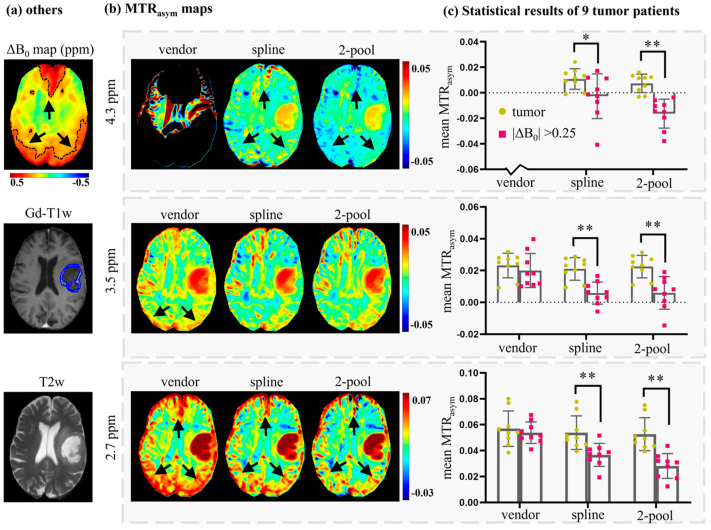
A representative brain tumor patient (**a**,**b**) and the statistical results of nine brain tumor patients (**c**). (**a**) ROIs of high B_0_ inhomogeneity regions (overlapped on ΔB_0_ map), ROI of tumor (overlapped on Gd-T1w) and the corresponding T2w. (**b**) MTR_asym_ maps corrected using the vendor, spline, and 2-pool Lorentzian methods. (**c**) The statistical results of nine tumor patients, which compared tumor regions with high B_0_ inhomogeneity regions on MTR_asym_ corrected using the vendor, spline and 2-pool Lorentzian methods. Because the vendor filtered many voxels of MTR_asym_ (4.3 ppm), we excluded MTR_asym_ (4.3 ppm) provided by the vendor. * *p* < 0.05; ** *p* < 0.01.

To further evaluate the effects of B_0_ inhomogeneity on tumor analysis, we compared the mean MTR_asym_ values of tumor ROIs and high ΔB_0_ ROIs (|ΔB_0_|>0.25 ppm), corrected using the vendor, spline and two-pool Lorentzian methods. Tumor ROIs were annotated on Gd-T1w by one experienced radiologist, and high ΔB_0_ ROIs were generated via threshold segmentation with threshold values = 0.25 ppm. Figure 6a shows the tumor ROI (overlapped on Gd-T1w), high ΔB_0_ ROI (overlapped on ΔB_0_ map) and T2w of another representative patient. As seen in Figure 5b and Figure 6b, MTR_asym_ (4.3 ppm) corrected using the vendor filtered too many voxels; therefore, we excluded it from the statistical analysis. Figure 6c shows that MTR_asym_ corrected using the vendor could not differentiate tumors from B_0_ artifacts, while the spline method and our two-pool Lorentzian method could efficiently reduce B_0_ artifacts, which enabled tumors and B_0_ artifacts to be distinguished (*p* < 0.05). The mean MTR_asym_ values of high ΔB_0_ ROIs corrected using our two-pool Lorentzian method were slightly lower than those obtained using the spline method at 2.7 and 4.3 ppm, which may have been due to the reduced B_0_ artifacts, such as the regions indicated by black arrows in Figure 2, Figure 5b and Figure 6b.

## 5. Discussion

In this study, to improve B_0_ correction performance and fully use all the acquisition offsets, we proposed a practical B_0_ correction approach for the most popular six-offset acquisition APT protocol. This approach employed a two-pool (symmetric water and asymmetric solutes) Lorentzian line to fit the Z-spectral shape of human brains at 3T with B_1_ = 2 μT, in a voxel-by-voxel manner (Figure 1). We evaluated our method through two kinds of experiments. Firstly, to validate the accuracy of our method, we acquired densely sampled 16-offset APT data of eight subjects, using its spline-interpolation correction results as the gold standard. As suggested by this experiment, and especially by the statistical results, the use of our method reduced MTR_asym_ errors more efficiently than the spline-interpolation-based method and one-pool-Lorentzian-based method (*p* < 0.01). For 4.3 ppm, the error of Z-spectra and MTR_asym_ corrected using our method were almost half of the errors caused by the spline and one-pool Lorentzian methods (Figure 2, Figure 3 and Figure 4). Secondly, for comparison with the vendor, we recruited nine brain tumor patients, from whom we acquired six-offset APT data. The experimental results suggested that our two-pool Lorentzian methods efficiently reduced B_0_ artifacts, which enabled tumor regions and B_0_ artifacts to be distinguished (*p* < 0.01), while the vendor could not differentiate tumors from artifacts (Figure 5 and Figure 6).

Interpolation-based methods are very commonly used in B_0_ correction. Debnath et al. compared the B_0_ correction performance of different interpolation algorithms with different step sizes on 64-offset APT data (B_0_ = 3T, B_1_ = 2 μT), and found that linear interpolation-based methods were suitable [15]. As seen in Figure S1, the performance of different interpolation-based methods (linear, cubic-Hermite, and cubic-spline) were similar when using 16 densely sampled offsets. However, for the sparse acquisition scenario (i.e., six offsets), linear and cubic-Hermite showed severe B_0_ artifacts on MTR_asym_ (2.7 ppm); cubic-spline (spline) outperformed these two algorithms but still displayed B_0_ artifacts. This may suggest that we need to choose interpolation algorithms for B_0_ correction with caution when sampled offsets are few, and spline interpolation may be suitable.

The step size of 6-offset APT data is the same with 16-offset data, but 6-offset acquisition does not cover the entire Z-spectrum. Windschuh et al. and Stancanello et al. also indicated that a full Z-spectrum is important for B_0_ correction [12,28]. These results may suggest that Z-spectral line shapes are necessary for accurate B_0_ correction. Zhou et al. reported that the Z-spectral line shape under 3T with high B_1_ is determined via the symmetric effect around water frequency (i.e., direct water saturation) and asymmetric effects (i.e., MT, NOE and CEST) [9,24]. Lee et al. separated Z-spectra into symmetric and asymmetric components for analysis and used a Lorentzian model to fit the symmetric component [23]. In addition, the broadening Z-spectra under 3T with high power did not show specific “visible” peaks. Therefore, a two-pool (symmetric water and asymmetric solutes) Lorentzian model may accurately describe the Z-spectral line shape, which could provide important a priori line-shape information for the B_0_ correction of six-offset APT data. As seen in Figure 3, using the Z-spectra of regions with low B_0_ inhomogeneity as the internal standard, we found that the Z-spectral line shapes corrected using our method were similar to the internal standard, while those corrected using the spline-based method were determined by acquired data, and those corrected using the one-pool-Lorentzian-based method were too symmetric (Figure 3). The statistical analysis of Z-spectra errors in eight subjects also demonstrated that our method was close to the gold standard and reduced errors more effectively than the spline and one-pool Lorentzian methods (Figure 4).

The proposed method could alleviate the analysis errors caused by B_0_ inhomogeneity and could be combined with CEST analysis methods to provide more metabolic information. For example, in this study, we provided more MTR_asym_ maps with higher image quality and less B_0_ artifacts than the vendor (Figure 5 and Figure 6), i.e., MTR_asym_ (2.7 ppm) reflecting fast exchange amide, including glutamate and MTR_asym_ (4.3 ppm) reflecting semi-solid MT components. Glutamate is an important energy source for tumor cells, and it always appears in tumor cells that are rapidly growing and dividing [29,30]. In addition, it is a biomarker for the diagnosis and assessment of various psychiatric and neurological disorders [31,32]. MT reflects myelin integrity and, to a lesser extent, cell membrane integrity, which could be used as a biomarker for neurological diseases in which the myelination of the brain is altered, such as in multiple sclerosis [33,34]. Moreover, Mehrabian et al. also found that MT is sensitive to treatment-induced changes in glioblastomas [35].

As the next step, we will combine our method with more CEST analysis methods to provide a comprehensively metabolic delineation of brain tumors. In addition to MTR_asym_, CEST frequency importance analysis could provide more metabolic features of all the acquired offsets, including upfield NOE offsets [36]. We will also combine our B_0_ correction method with the CEST frequency importance analysis method to fully use all the acquired offsets.

Although our method showed better performance than the interpolation-based method, some limitations remain. (1) In this study, we employed 17 subjects to demonstrate the performance of our method; however, for clinical application, our findings need to be supported by experimental results from studies that have used a larger number of subjects. We will collect more data to evaluate the proposed method more comprehensively. (2) A drawback of the Lorentzian fitting method is the relatively lengthy computation time required. Due to the voxel-by-voxel correction, the computation time of our method was about 7 min for one subject. (3) The Lorentzian fitting method is sensitive to the starting points and boundaries of parameters, and there is not a common method used to determine appropriate starting points and boundaries. Meanwhile, the choice of parameter boundaries influences the fitting time. (4) Limited by the small number of acquisition offsets, we used a simplified two-pool Lorentzian model to replace the multiple-pool model. Fortunately, some studies have focused on accelerating Lorentzian fitting. For example, Yao et al. classified voxels into several clusters, and only conducted Lorentzian fitting once for a cluster to reduce the fitting time [22]. Zaiss et al. employed neural networks to predict the parameters of Lorentzian function, and accelerated Lorentzian fitting to several seconds [37]. In this study, we demonstrated the feasibility of our method. As the next step, we will combine our method, neural networks and densely sampled simulation data, which will enable us to realize a quick multiple-pool Lorentzian-based correction method without tuning fitting parameters.

## 6. Conclusions

In this study, a practical B_0_-correction approach is proposed, which employed a simplified two-pool Lorentzian model for Z-spectral fitting with B_1_ ≥ 1 μT under 3T. For both healthy subjects and tumor patients, our approach outperformed conventional interpolation, allowing for better correction at limbic offsets. Therefore, this approach may allow for the efficient extraction of CEST contrast at multiple frequency offsets, and facilitate the more comprehensive metabolic analysis of brain tumors.

## Figures and Tables

**Table 1:** Starting points and boundaries of the 2-pool Lorentzian fitting parameters.

	Δ_1_	*W*_1_	*A*_1_	Δ_2_	*W*_2_	*A*_2_	*Z_base_*
start	0	50	25	−2	50	0.1	0.7
lower	−0.5	0	0	−4	−inf	−inf	−inf
upper	0.5	100	50	4	+inf	+inf	+inf

Note: +inf and −inf represent the positive and negative infinity, respectively.

## Data Availability

The human data used in this study cannot be shared at this time as the data also form part of an ongoing study.

## Supplementary Materials

The following supporting information can be downloaded at: https://www.mdpi.com/article/10.3390/tomography8040165/s1, Figure S1. MTR_asym_ maps corrected employing three interpolation-based methods (i.e., linear, cubic-Hermiter, and cubic-spline), using 16 offsets and 6 offsets, respectively. Figure S2. MTR_asym_ maps and MTR_asym_ error maps of a representative healthy volunteer. (a) MTR_asym_ maps without B_0_ correction, gold standard and MTR_asym_ maps corrected using spline, 1-pool Lorentzian and 2-pool Lorentzian methods; (b) T2w, Gd-T1w and ΔB_0_ map; (c) the corresponding MTR_asym_ error maps with gold standard. Figure S3. ROI analysis of a representative healthy volunteer. (a) Four circle ROIs (radius = 5 voxels) with different B_0_ inhomogeneities, shown on T2w and ΔB_0_ map; (b) mean Z-spectra of ROIs, including gold standard, and Z-spectra corrected using spline, 1-pool Lorentzian, and 2-pool Lorentzian methods; (c) the corresponding Z-spectra errors with gold standard.

## Abbreviations

APT	amide proton transfer
MTR_asym_	asymmetry analysis of magnetization transfer ratio
CEST	chemical exchange saturation transfer
ΔB_0_	B_0_ inhomogeneity
WASSR	water saturation shift referencing
DS	direct water saturation
MT	magnetization transfer
NOE	nuclear Overhauser effect
Δ*_i_*	central offset of the Lorentzian line
FWHM	peak full width at half maximum
*A_i_*	peak amplitude
ROI	region-of-interest
