# Supplementary material for: B0 Correction for 3T Amide Proton Transfer (APT) MRI Using a Simplified Two-Pool Lorentzian Model of Symmetric Water and Asymmetric Solutes
Source: Tomography. 2022 Aug 1;8(4):1974–86. doi: 10.3390/tomography8040165 (PMC9412582; doi:10.3390/tomography8040165)
Supplement: Supplementary file 1 [file tomography-08-00165-s001.zip › tomography-1734322-supplementary.pdf]

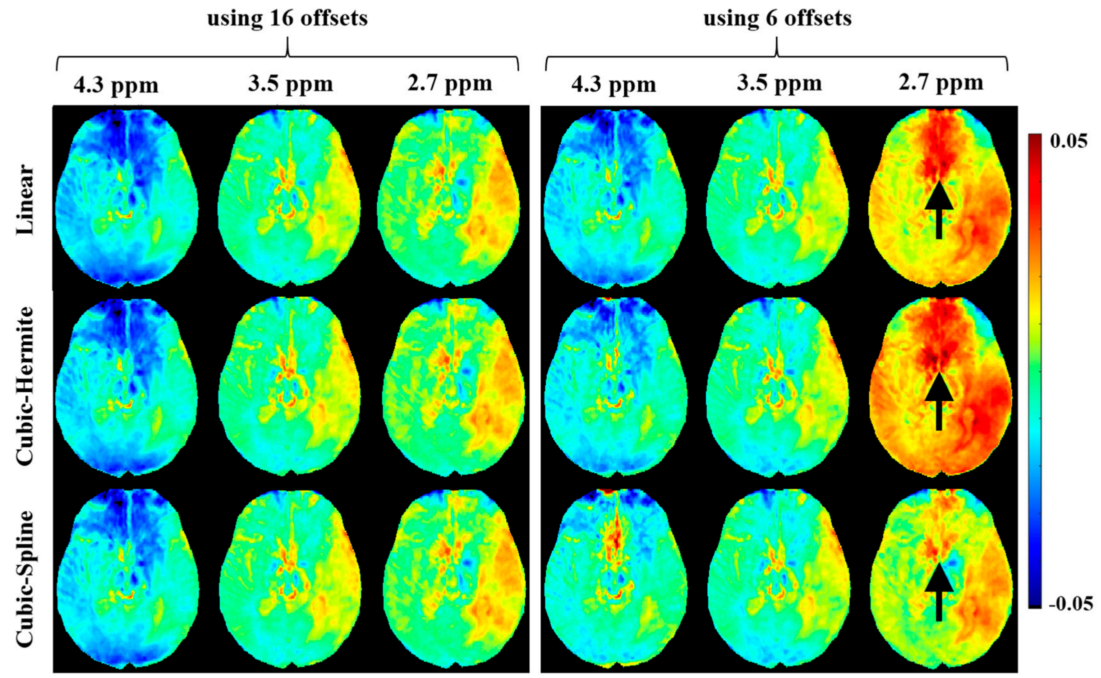

Figure S1. MTR_{asym} maps corrected employing three interpolation-based methods (i.e., linear, cubic-Hermiter, and cubic-spline), using 16 offsets and 6 offsets, respectively.

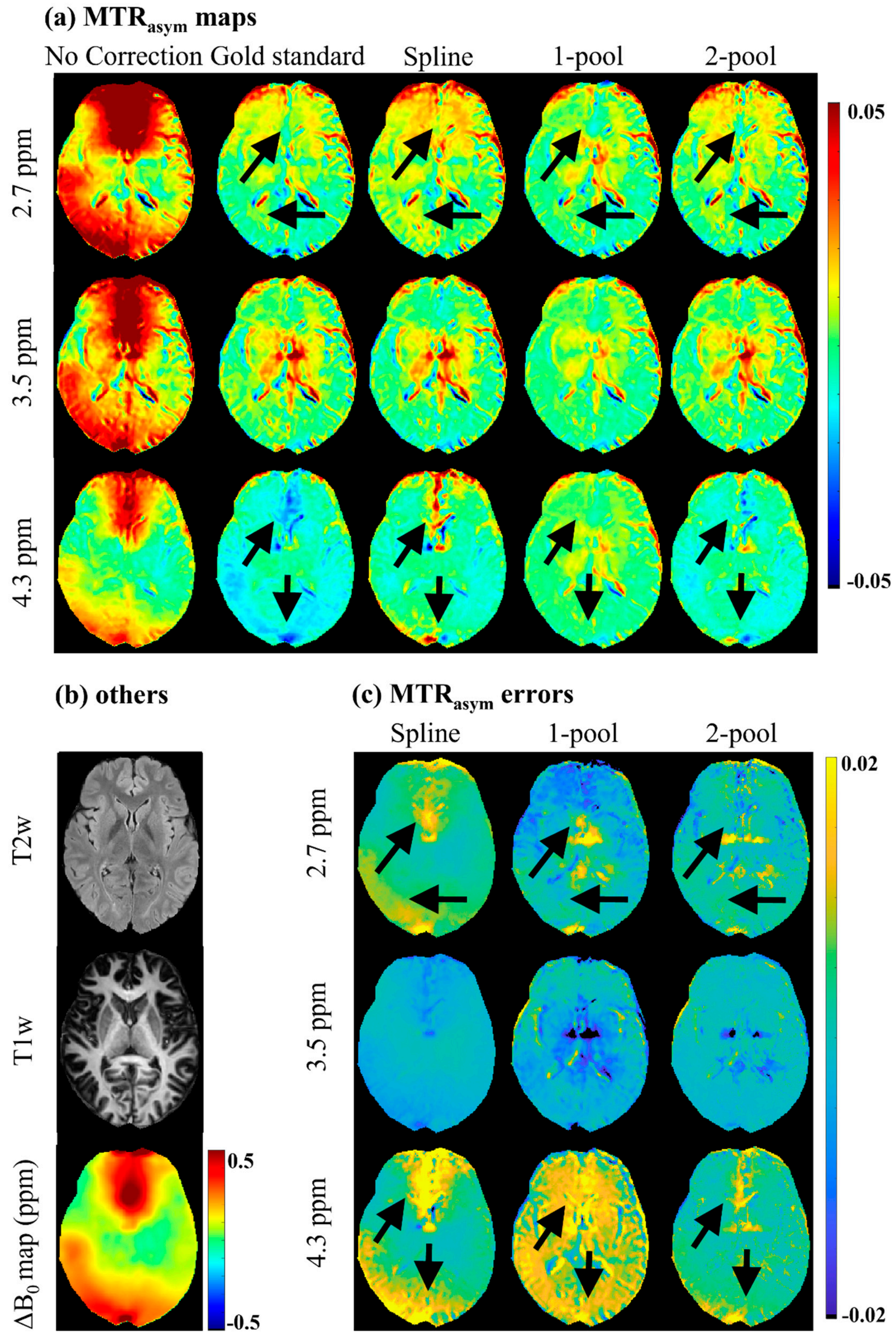

Figure S2. MTR$_{asym}$ maps and MTR$_{asym}$ error maps of a representative healthy volunteer. (a) MTR$_{asym}$ maps without B_0 correction, gold standard and MTR$_{asym}$ maps corrected using spline, 1-pool Lorentzian and 2-pool Lorentzian methods; (b) T2w, Gd-T1w and ΔB_0 map; (c) the corresponding MTR$_{asym}$ error maps with gold standard.

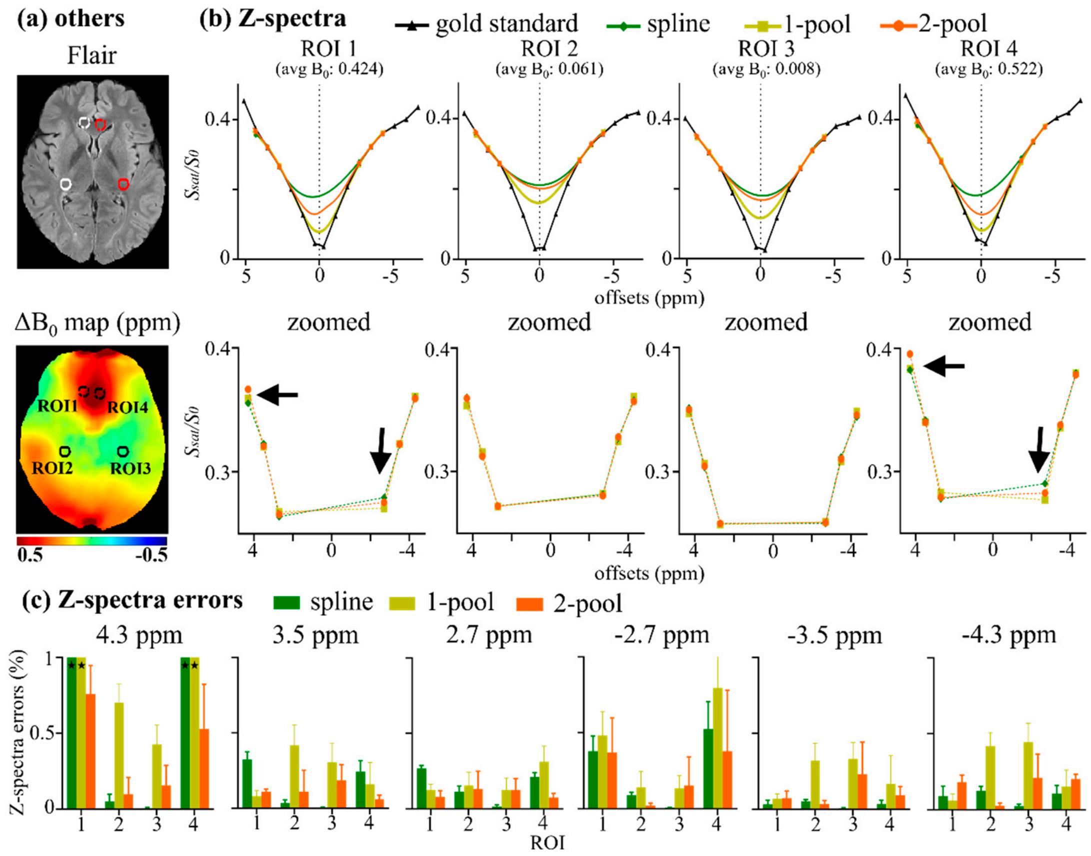

Figure S3. ROI analysis of a representative healthy volunteer. (a) Four circle ROIs (radius = 5 voxels) with different B_0 inhomogeneities, shown on T2w and ΔB_0 map; (b) mean Z-spectra of ROIs, including gold standard, and Z-spectra corrected using spline, 1-pool Lorentzian, and 2-pool Lorentzian methods; (c) the corresponding Z-spectra errors with gold standard.
